# Qualitative interviews to evaluate content validity of the ACTIV-2 COVID-19 Symptom Diary (ACSD)

**DOI:** 10.1186/s41687-022-00535-x

**Published:** 2023-01-31

**Authors:** Louis S. Matza, Katie D. Stewart, April N. Naegeli, Kayla M. Mills, Karin S. Coyne, Kara W. Chew, Michael D. Hughes, Davey M. Smith

**Affiliations:** 1 Evidera, Bethesda, MD USA; 2 Eli Lilly and Company, Indianapolis, IN USA; 3 Formerly of Eli Lilly and Company, Indianapolis, IN USA; 4 David Geffen School of Medicine at UCLA, Los Angeles, CA USA; 5 Harvard T.H. Chan School of Public Health, Boston, MA USA; 6 University of California San Diego, San Diego, CA USA

## Abstract

**Background:**

Patient-reported outcome measures are needed to assess the impact of treatments for COVID-19 on symptoms. The ACTIV-2 COVID-19 Symptom Diary (ACSD) is being used in the ongoing Accelerating COVID-19 Therapeutic Interventions and Vaccines-2 (ACTIV-2) platform clinical trial. The purpose of the current study was to conduct qualitative interviews to assess content validity of the ACSD.

**Methods:**

Interviews were conducted with adults who had tested positive for SARS-CoV-2. The ACSD begins with global items, followed by a symptom checklist. Each interview began with concept elicitation focusing on participant experiences with COVID-19. Then, participants completed the ACSD, and cognitive interviews were conducted to evaluate the questionnaire. Interviews were recorded, transcribed, and coded following a qualitative content analysis. For the qualitative analysis, a coding dictionary was developed with a list of all potential codes and instructions for how the codes should be applied and combined.

**Results:**

Interviews were conducted with 30 participants (mean age = 39 years; 57% female; 17% Latinx; 17% Black/African American; 40% meeting at least one criterion for classification as high risk of progression to severe COVID-19). Commonly reported symptoms included fatigue (reported by 100% of the sample), body pain/muscle pain/aches (87%), headaches (87%), cough (83%), loss of smell (73%), shortness of breath/difficulty breathing (70%), and chills (70%). The 13 symptoms most commonly reported in this study are included in the ACSD. After completing the ACSD, participants consistently reported that it was clear and easy to complete, and all items were generally interpreted as intended. Based on participants’ input, the ACSD was edited slightly after the first 13 interviews, and the revised version was used for the final 17 interviews. Two additional items assessing “brain fog” and dizziness were recommended for addition to the ACSD in future research.

**Conclusions:**

This qualitative study supports the content validity of the ACSD for assessment of COVID-19 symptoms. Quantitative research with larger samples will be needed to examine the questionnaire’s measurement properties.

**Supplementary Information:**

The online version contains supplementary material available at 10.1186/s41687-022-00535-x.

## Plain English summary

This study focused on the ACTIV-2 COVID-19 Symptom Diary (ACSD), a questionnaire that assesses symptom severity of COVID-19. The ACSD begins with global items assessing overall symptom severity, followed by a symptom checklist focusing on individual symptoms. Interviews were conducted with 30 adults who had tested positive for COVID-19. The patients reported their experiences with COVID-19, completed the ACSD, and provided their opinions about the ACSD. Based on input from these patients, the ACSD appears to be clear and easy to complete, and it includes the most common and important symptoms of COVID-19. The ACSD was edited for clarity, and “brain fog” and dizziness were recommended additions for future research. This study suggests that the ACSD is a useful questionnaire for assessment of COVID-19 symptoms in clinical studies. Studies like this are important for ensuring that symptoms are measured appropriately and accurately in clinical trials. Future research with larger samples will be needed to further examine the questionnaire.

**Supplementary Information:**

The online version contains supplementary material available at 10.1186/s41687-022-00535-x.

## Introduction

In late 2019, a novel coronavirus, severe acute respiratory syndrome coronavirus 2 (SARS-CoV-2), quickly began to spread through the human population, resulting in the coronavirus disease 2019 (COVID-19) pandemic [1, 2]. Several safe and effective vaccines for COVID-19 are currently available [3]. In addition, a range of treatments are currently under investigation, including monoclonal antibodies and antivirals [4–8]. As treatments for COVID-19 are developed, patient-reported outcome (PRO) measures are needed to assess their impact on symptoms.

In persons with COVID-19, PRO measures could be used to assess treatment efficacy and track symptoms [9, 10]. PRO measures assessing COVID-19 symptoms are particularly important in phase 3 studies, for which the Food and Drug Administration (FDA) has recommended endpoints providing an indication of “how a patient feels, functions, or survives” rather than virologic endpoints [11]. PRO development and validation typically requires several years of qualitative and quantitative research before a questionnaire can be considered valid and fit-for-purpose in a clinical trial [12–14]. However, because of the urgency of the COVID-19 pandemic and the immediate need for effective treatments, clinical trials were planned and initiated at an unusually fast pace. Therefore, symptom measures had to be included in these trials before they could be validated for use in the target population.

Some trials of treatments for COVID-19 used PRO measures that had been validated for use in other medical conditions with similar symptoms to COVID-19, such as the Influenza Patient-Reported Outcome (FLU-PRO), which was developed for assessment of influenza symptoms [15, 16]. In some cases, previously existing PRO measures were adapted to COVID-19 based on clinical impressions of typical COVID-19 symptoms. For example, items querying loss of taste and smell were added to the original Flu-PRO to create the Flu-PRO Plus for use with persons with COVID-19 [17]. While these measures have been useful, the relevance, comprehensiveness, and validity for use in persons with COVID-19 are uncertain.

For other trials, new questionnaires were drafted specifically for the purpose of assessing symptoms of COVID-19. One of these questionnaires was developed for use in the Accelerating COVID-19 Therapeutic Interventions and Vaccines-2 (ACTIV-2) phase 2/3 platform trial [18–20]. In ACTIV-2, investigational agents are initially studied in a phase 2 evaluation. Promising investigational agents can move on to phase 3 evaluation within the same platform, allowing for rapid evaluation of multiple therapies for outpatients with COVID-19. The newly developed PRO, called the ACTIV-2 COVID-19 Symptom Diary (ACSD), was developed by the clinical trial team as a daily diary to assess COVID-19 symptom severity.

The current qualitative study was initiated after the ACTIV-2 trial had begun. The purpose of this study was to conduct interviews with COVID-19 outpatients to assess the content validity of the ACSD. Content validity is the extent to which an instrument has the relevant and important aspects of the concept it was designed to measure [21], and it is documented primarily via qualitative research with individuals from the target population [12–14, 22].

## Methods

### Overview of study design

In this qualitative study, one-on-one interviews were conducted by telephone with adult outpatients who had tested positive for SARS-CoV-2. Each interview lasted approximately 60–90 min including (1) concept elicitation focusing on participants’ experiences with COVID-19, (2) completion of the ACSD, and (3) cognitive interviews to evaluate the ACSD. Interviews were recorded and transcribed, and transcripts were coded. Methods and materials were approved by an independent review board (Ethical and Independent Review Services [E&I], E&I study numbers 20151–01), and participants provided written informed consent prior to completing any study procedures.

Interviews were conducted in two phases between November 2020 and May 2021. After the first 13 interviews (phase 1), minor revisions were made to the ACSD based on feedback from participants. The revised version of the diary was used for the final 17 interviews (phase 2).

### Participants

Participants were recruited from four clinical sites in the US, located in Gainesville, GA, Jackson, TN, Guntersville, AL, and Miami, FL. All participants had a confirmed diagnosis of COVID-19 based on a positive SARS-CoV-2 nucleic acid or antigen test and experienced symptoms consistent with COVID-19 within the seven days prior to screening. Participants were required to read and speak English. Efforts were made to recruit a sample with approximately equal gender distribution and a balance between participants at high and low risk for developing severe COVID-19. Based on previous studies of disease progression [4, 23], participants were considered high risk if they were ≥ 55 years of age and/or had one of the following conditions: chronic lung disease, moderate-to-severe asthma, obesity (body mass index > 35 kg/m^2^), hypertension, cardiovascular disease (including history of stroke), diabetes, chronic kidney disease, or chronic liver disease [4, 23]. Additionally, efforts were made to recruit a sample that was racially diverse based on the racial breakdown of COVID-19 cases in the US population [24].

### Measures

#### ACTIV-2 COVID-19 Symptom Diary (ACSD)

The ACSD was developed in early 2020 to assess COVID-19 symptoms. The items were drafted based on the symptoms that clinicians (who are all part of the ACTIV-2 trial team) commonly observed among patients with COVID-19 during the initial months of the pandemic, consideration of measures used to assess influenza [15, 16, 25, 26], and early reports of COVID-19 from China [23]. The ACSD was designed to be completed daily by outpatient adults with mild-to-moderate COVID-19 participating in phase 2 and phase 3 trials. The diary begins with a series of global items, followed by a checklist of symptoms rated on a four-level severity scale (absent, mild, moderate, severe), and two dichotomous items (yes, no) assessing loss of taste and smell.

**Fig. 1:**
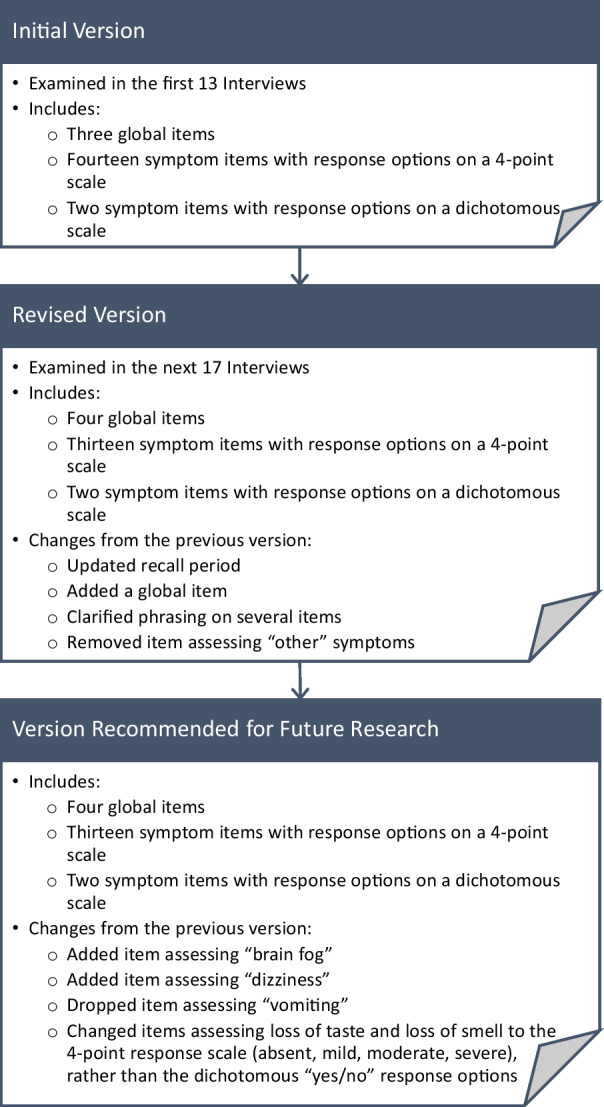
Versions of the ACTIV-2 COVID-19 Symptom Diary (ACSD)

Three versions of the ACSD are discussed: the initial version, the revised version, and a version recommended for future research. The first phase of interviews (n = 13) was conducted using the “initial version” (presented in Additional file 1). The “revised version” (Appendix A) was administered in the second phase (n = 17). The revised version includes minor revisions based on feedback from the first 13 interviews and decisions made by the clinical trial team. Based on qualitative analysis of data from all 30 interviews, four additional edits were made in the “version recommended for future research” (Appendix B). Figure 1 summarizes these three versions of the ACSD.

#### Sociodemographic form

Participants completed a sociodemographic form that included items on age, gender, living situation, employment, education level, racial/ethnic background, and general health-related questions including one about comorbid conditions. To limit the transmission of COVID-19 during the pandemic, participants did not mail their completed study materials to the study team. Instead, the participants dictated their responses to the interviewers on the phone after completing the sociodemographic form, with the interviewers transcribing the dictated responses.

#### Qualitative interview guide

Interviews were conducted following a semi-structured interview guide, beginning with a concept elicitation discussion designed to elicit descriptions of COVID-19 symptoms. Participants were asked what they experienced with COVID-19, when their symptoms began, how their COVID-19 started, the first things they noticed, and which symptoms were currently the most bothersome. They were also asked which symptoms had been the most bothersome since they were diagnosed. These open-ended questions were designed to elicit spontaneous reports of symptoms before the interviewer probed for discussion of other common symptoms not yet mentioned by the participant.

After completing the concept elicitation, the interviewer instructed the participant to complete the ACSD independently (“Please open the envelope that was mailed to you and take out the Participant Study Diary. Please complete the diary and let me know when you are finished”). Then, the interview proceeded with a debriefing on the ACSD to assess the ease of completion and comprehensiveness of the questionnaire, as well as relevance, clarity, and interpretation of the items. Participants were asked to explain their interpretation of the instructions, response options, and recall period. For each item on the ACSD, participants explained in their own words what the item was asking, if any items were difficult to understand or answer, how easy it was to respond to the item, which response they selected, and why.

### Interview and analysis procedures

Interviews were conducted by telephone by four trained interviewers following the semi-structured interview guide. All interviews were audio recorded and professionally transcribed.

The interview transcriptions from phases 1 and 2 were analyzed together following a qualitative content analysis approach [27] using ATLAS.ti (version 8), a software designed for systematic analysis of qualitative data. A coding dictionary was developed based on the interview guide as well as themes and concepts that emerged during the interviews. This document provided a list of all potential codes with the definition of each code and instructions for how each code should be applied and combined with other codes. Statements in the transcripts were coded based on this dictionary and grouped into key concepts. All coders had received prior training in qualitative analysis theory and practice, in addition to a study-specific training on the coding dictionary. Three staff members independently coded the first interview transcript, their codes were compared, discussed, and reconciled wherever there were differences. After agreement was reached and the analysis team was confident that the coders were applying the codes consistently, the three coders independently coded the remaining transcripts.

The coded text from the concept elicitation portion of the interview resulted in qualitative output that identified participants’ statements and categorized them according to the concepts that were discussed. The analysis of data from the cognitive interview portion included assessment of (1) the clarity of the questionnaire items; (2) how respondents interpreted the items; (3) difficulty completing items; (4) the comprehensiveness of the instrument; and (5) the appropriateness of the response scales and recall periods used in the instrument. The coded data were used to develop a saturation grid to document the frequency of participants with statements corresponding to each concept. Saturation was reached when no new information was gained from additional interviews [28].

## Results

### Participant characteristics

Thirty participants completed the interviews (Table 1). The sample had a mean age of 39.4 years and was 57% female, 17% Latinx, 17% Black/African American, and 80% White. The sample was diverse with regard to educational status, including a total of 17 participants (56.7%) who did not have a college degree. Twelve participants met at least one criterion for classification as high risk of progression to severe COVID-19 (≥ 55 years old [n = 3], obesity [n = 6], diabetes [n = 4], hypertension [n = 2], cardiovascular disease [n = 1], chronic kidney disease [n = 1]). Over half of participants (n = 17; 57%) reported having no comorbid health conditions. The comorbid conditions reported by the most participants were obesity (n = 6; 20%), anxiety (n = 6; 20%), and diabetes (n = 4; 13%). The sample varied widely in the time since onset of COVID-19 symptoms. Of the 30 participants, 25 were interviewed less than 30 days following symptom onset, while five were interviewed more than 30 days after they first had symptoms (i.e., 43, 77, 81, 99, and 158 days later). These five all reported having current symptoms.

**Table 1:** Sample characteristics (N = 30)

Characteristics	Descriptive statistics
Age (Mean, SD)	39.4 (12.3)
Gender (n, %)	
Male	13 (43%)
Female	17 (57%)
Ethnicity (n, %)	
Hispanic or Latino	5 (17%)
Not Hispanic or Latino	25 (83%)
Race (n, %)	
Asian	1 (3%)
Black or African American	5 (17%)
White	24 (80%)
Employment status (n, %)	
Full-time work	21 (70%)
Part-time work	6 (20%)
Other^1^	3 (10%)
Highest level of education completed (n, %)	
Secondary, high school, or GED	12 (40%)
Associate degree, technical, or trade school	5 (17%)
College or university degree (BA, BS)	10 (33%)
Post-graduate degree (MA, MBA, PhD)	3 (10%)
Marital status (n, %)	
Single	7 (23%)
Married/Cohabitating/Living with partner	19 (63%)
Other^2^	4 (13%)
Time since onset of COVID-19 symptoms^3^	
Mean days (SD)	28.1 (33.3)
Median days [Range]	16.5 (6.0–158.0)
Time since positive test for COVID-19	
Mean days (SD)	26.5 (33.4)
Median days (Range)	15.0 (5.0–157.0)

^1^Other employment includes: unemployed (n = 2) and not specified (n = 1)

^2^Other marital status includes: divorced (n = 2), separated (n = 1), and widowed (n = 1)

^3^There were five participants whose interview occurred more than 30 days following the onset of symptoms, with durations of 43, 77, 81, 99, and 158 days. All five of these participants reported having current symptoms at the time the interview occurred. All other interviews occurred less than 30 days following symptom onset

### Concept elicitation

The wide range of symptoms reported by participants during concept elicitation highlights the heterogeneity of COVID-19 (Table 2). The most common symptoms included fatigue (100% of the sample), body pain/muscle pain/aches (87%), headaches (87%), cough (83%), loss of smell (73%), shortness of breath/difficulty breathing (70%), chills (70%), loss of taste (67%), and nasal congestion (67%). Even among these common symptoms, participant descriptions suggest that individual experiences were heterogeneous. For example, some participants described their cough as “productive” while others said it was “dry.” Many participants described “aches” or “pains” in their body, while others reported “joint pain” or that their “bones hurt.” Body aches/pain, which tended to last a few days, was the most common symptom mentioned when participants were asked which symptoms were most bothersome. Fatigue tended to be long-lasting (i.e., several reports of over a week in duration), but participants reported fluctuations in fatigue severity throughout the day or from day to day.

**Table 2:** COVID-19 Symptoms Reported by Participants (N = 30)

Symptoms	Total number of participants reporting each symptom	Participants who reported each symptom spontaneously^1^	Participants who reported each symptom in response to interviewer probe^2^	Participants who reported each symptom as one of the most bothersome^3^
	n (%)	n (%)	n (%)	n (%)
*Symptoms included on the revised version of the ACSD*				
Cough	25 (83%)	15 (50%)	10 (33%)	4 (13%)
Shortness of breath or difficulty breathing	21 (70%)	12 (40%)	9 (30%)	5 (17%)
Feeling feverish	14 (47%)	1 (3%)	13 (43%)	-
Chills	21 (70%)	5 (17%)	16 (53%)	-
Fatigue (low energy)	30 (100%)	24 (80%)	6 (20%)	9 (30%)
Body pain or muscle pain or aches	26 (87%)	18 (60%)	8 (27%)	10 (33%)
Diarrhea	18 (60%)	5 (17%)	13 (43%)	1 (3%)
Nausea	18 (60%)	8 (27%)	10 (33%)	2 (7%)
Vomiting^4^	4 (13%)	4 (13%)	-	1 (3%)
Headaches	26 (87%)	22 (73%)	4 (13%)	6 (20%)
Sore throat	19 (63%)	9 (30%)	10 (33%)	-
Nasal obstruction or congestion (stuffy nose)	20 (67%)	9 (30%)	11 (37%)	3 (10%)
Nasal discharge (runny nose)	18 (60%)	4 (13%)	14 (47%)	1 (3%)
Loss of taste^4^	20 (67%)	15 (50%)	5 (17%)	6 (20%)
Loss of smell^4^	22 (73%)	19 (63%)	3 (10%)	7 (23%)
*Other symptoms reported by participants*^*5*^				
Fever	15 (50%)	14 (47%)	1 (3%)	1 (3%)
Confusion/Brain fog^4^	8 (27%)	8 (27%)	-	1 (3%)
Dizziness^4^	8 (27%)	8 (27%)	-	1 (3%)
Weakness	6 (20%)	6 (20%)	-	2 (7%)
Lack of appetite	5 (17%)	5 (17%)	-	-
Chest tightness	4 (13%)	4 (13%)	-	-
Nasal/Sinus drainage	3 (10%)	3 (10%)	-	1 (3%)

^1^Symptoms that were introduced by the participant, without prompting from the interviewer, were coded as “spontaneous.”

^2^If a participant did not spontaneously report a symptom that appears on the ACSD, the interviewer asked about the symptom. If participants responded by indicating that they had experienced the symptom, it was coded as “probed.”

^3^Statistics in this column are based on responses to the question “Since you were diagnosed with COVID-19, which symptoms have been most bothersome for you?”

^4^Four changes to the ACSD are recommended for future research: dropping the items assessing vomiting, adding items assessing brain fog and dizziness, and using 4-point response scales (i.e., absent, mild, moderate, severe) for loss of taste and smell rather than dichotomous responses (i.e., yes, no)

^5^The rows of this table include all symptoms reported by at least three of the 30 participants. Symptoms reported by only two participants included chest congestion, eye symptoms other than vision, chest pain, swollen glands/lymph nodes, burning in sinus cavity/nose, elevated heart rate, pneumonia, and blood clots in lungs. Symptoms reported by only one participant included heart palpitations, ear pain, ear ringing/tinnitus, chest pressure, autoimmune flare-up, throat tickle, inflammation, eczema, swollen ankles, frequent urination, loss of sleep, swelling in cheeks/face, drop in oxygen levels, increased blood pressure, muscle cramps, nose bleeds, clogged ears, feeling like [they are] going to pass out, feeling hot and sweaty, lightheaded, feeling run down, stiffness, joint swelling/inflammation, constipation, loss of muscle mass, feeling like [they are] going to get a headache, rash, spots on lips, swollen tongue, ulcers, and blurred vision

To describe dyspnea, participants typically used the phrases “shortness of breath,” “difficulty breathing,” “feeling winded,” or “unable to catch my breath.” A typical dyspnea episode lasted a few minutes and was brought on by activity, and this symptom usually recurred for over a week. Some participants reported constant shortness of breath, even at rest. Most participants who reported loss of smell experienced a complete loss of smell at some point, but they also described a partial return of their sense of smell in terms of percentage of smell or ability to smell certain scents. Others described an altered or distorted sense of smell as “chemical,” “metallic,” or “musky.” When discussing nasal congestion, nearly all used the term “congestion,” but a few referred to a “stuffy nose” or being “stuffed up.” Participants’ descriptions of the most reported symptoms are presented in Table 3.

**Table 3:** Selected Quotations: Patients’ Descriptions of Common COVID-19 Symptoms

Symptom	Selected Quotations
Cough	• **It was dry, it was never productive**, and that was it. **It wasn’t consistent**, like there wasn’t a time during the day that I would start coughing and couldn't stop, it was never like that. It was just like random times during the day. • It was a **continual cough**. I felt like I had a tickle in my throat…and then it went to like a deep cough. It's almost like I couldn’t catch my breath because I just kept coughing all day and it was horrible because I was working.
Shortness of breath or difficulty breathing	• **I couldn’t catch my breath**. I remember I was eating a sandwich and I kept trying to gulp for air whilst eating and I felt like I could just die. It was just, it was just terrible. **It's like I wasn’t getting enough air** in and I was gasping for air. • I can walk from my bedroom to the kitchen, and that's pretty much all that I could do, then **I have to take a breath**, then I can proceed, just like I can make 20 to 25 steps, **I have to stop and breathe, then proceed, then stop and breathe, then proceed**. • I wasn’t able to sleep laying down flat **because I felt like I couldn't breathe**, so I was having to lay on my side and on my stomach, and that lasted for about a week-and-a-half from that point. • I had **a really hard time breathing**, like I felt like sometimes I was often having a panic attack because **I felt like I couldn't catch my breath**.
Feeling feverish	• **I would just be cold** and like I wouldn't even be hot to touch, like most of the time with a fever when somebody has a fever, you can touch them and you’re like “ooh, yes, you have a fever,” but when **I was feeling feverish, I would be cold but you could touch me and I would not be hot**. • You **get cold, can’t get warm**, and your **body starts aching** and your muscles start aching. You feel **lethargic, irritated**. • I felt **warm and weak and tired**.
Chills	• I had **chills several times**, like you could actually see **goosebumps** and I would **feel cold**. I checked my temperature several times a day initially and I never recorded a temperature or fever…. • I **got very like cold** when it was hot, maybe like what they say the chills, like I **needed to cover myself** because I was extremely cold.
Fatigue (low energy)	• I pretty much was **sleeping the majority of the day**. The first two days that were the worst, I probably was only out of bed for maybe three hours out of those two days. Since then, things have gradually gotten a little bit better, but even a week ago I was having to take two or three naps a day. • If I do anything at all, it runs me down and then, you know, you just **don't have energy or don't want to do anything at all**. It just zaps your energy and you kind of, I don't know, you just **feel like a zombie** and you’re **tired all the time**, can’t think straight, like I said, no motivation at all or desire to do anything. • Yes, just **fatigued** and **low energy**, I couldn't hardly do anything.
Body pain or muscle pain or aches	• The body aches, **I just hurt all over**, it’s like I had been in a car accident or something, like I was just **sore all over**. • It was really like it **felt like my bones hurt**, like **every part from my head to my toes hurt constantly**. It was almost like a flu ache, and when I would run a fever, it would get worse, and like the covers being pulled over me or something like that would make it worse it seemed like. • **I ached everywhere**, I just kind of felt numb. The only way I know how to describe it is **my insides just hurt**.
Diarrhea	• I had to go to the bathroom, you know, in a hurry. And it's kind of liquid. • When I would go to the bathroom, it was like very loose, but it wasn’t like the entire time.
Nausea	• Just the **sensation of I’m about to throw up**… I mean the room wasn’t spinning or anything, it was just a sensation of oh, **I think I’m about to get sick**…. • It was just two or three days that I would cough so much or something to where it would just make me feel a **little nauseous**, and there was once that I recall thinking, oh my gosh, I’m going to throw up… I just had that **cottony feeling in my mouth**, but then once I lay down, kind of calmed down a little bit and tried to just get a cough drop to help my cough, then I felt better.
Vomiting	• Like when I tried to do something, I felt dizzy and I got short of breath, and one time **I think I overdo it and I vomited**, I vomited my guts out, I vomited like ten times. • It was rough, like I said, **every time I tried to eat, I would get sick**, so it was like coming out both ends, and if I did eat, I would throw up, so that was really the worst part of it.
Headaches	• I got that **horrible headache**. **I never thought my head could hurt like that**… I’ve never had a migraine, but it was so intense. • It **started out with a headache**, a pretty bad headache, I don't usually get headaches bad, and then I just sort of felt icky but it was mostly the headache. • I still have been having **headaches on and off** but they’re not like daily headaches but they’re still in the spot that I had a headache before. It’s didn't feel like a normal sinus headache or like a normal migraine, **it was like a weird headache**.
Sore throat	• It was just like a **scratchy throat**. • It’s kind of **achy** and it’s kind of **scratchy**. It’s **hard to swallow**, especially in the morning, it **hurts terribly** in the morning. • It was really **itchy** and felt like it **burned** a bit because I kept coughing and it made my **throat raw**.
Nasal obstruction or congestion (stuffy nose)	• I was completely **stuffed up**. • Your head is **congested** and the drainage that goes into your, you know, it drains down. That has been one of the worst things for me is the congestion… Because **every day constantly your head is congested** and the drainage is constantly there. • I did have **some congestion**, like I could feel that my nose was congested, but **I didn't have anything coming ou**t, like when I would blow my nose, it wasn’t anything, it just felt congested.
Nasal discharge (runny nose)	• I had a **runny nose**. • I had more congestion but **a lot of discharge four or five days**.
Loss of taste (dysgeusia)	• My food started **tasting weird**, and then I woke up the next morning and **I couldn't smell or taste anything**, and so I knew that that was like a big indicator. • I would take a drink of something that I knew what it was supposed to taste like and **I couldn't taste it**, or I would eat something that I knew should have a taste and I couldn't taste it. **There was nothing there**…. • That was really weird. I told the doctor it tasted like I had… **Metallic**. That's the word. Yes. It tasted—**things tasted metallic**…And **I couldn’t really taste anything** and I couldn’t smell anything. It was really weird. And food was just not enjoyable.
Loss of smell (anosmia)	• I can’t taste or smell. • I noticed that once I was spraying Lysol, you know, you could smell Lysol, **I couldn't smell it**, so I actually opened up a bottle of bleach and I tried to sniff that and I couldn't smell it. • The first thing was the **smell and it became distorted**, so it **really wasn’t like completely gone**… **I could smell, like it smelt like I had my nose in a bucket of rusty water or something**, it just had this [metallic] smell to it, kind of a burning smell I guess you could say, but that's what everything smelt like to me. I couldn't smell flowers and I couldn't even smell like rubbing alcohol or bleach, there was no smell like that, it was just **all that metallic smell that I smelt**.
Fever	• I had a **fever, a low-grade fever** from the very beginning, and it only went away like two weeks ago. • I had **low grade fever** for about three or four days, never getting over 100, and usually if I get a high fever.
Confusion/Brain fog	• I also had a real – and I feel like I’m finally coming out of that – a **brain fog that was just unreal**, I mean **I could not think clearly**, I could not remember anything…it was real intense I’d say for the first two full months. It was like you could not think clearly…I mean it’s like **I could not remember things, I could not focus**, I **could not concentrate** well, and I **felt like my head like I literally was in a fog**, it was the weirdest feeling ever. • I feel like my **processing speed like cognitively were really impaired**, and I think that is still a **lingering symptom**… I would say just overall cognition but like memory, processing speed, things like that are just at times they’re very delayed and it’s like you’re kind of cloudy.
Dizziness	• I also have like dizziness sometimes, like yesterday **I felt dizzy**, the day before yesterday I felt dizzy…it’s like the **balance thing**. It’s like just then I was standing, I’m sitting now, but a moment ago when I was talking to you, all of a sudden **I felt unsteady like I needed to lean on the counter**. • It’s almost like a **dizziness or a lightheadedness** but I wouldn't say that I feel that I’m spinning, like vertigo, but I will have spells that have gotten much less in severity and frequency now, but like two weeks ago there were some **multiple spells of almost feeling like you’re going to faint**, never that I would actually blacken out or I never felt like I couldn't walk or anything, it was almost like a **lightheaded sensation**.
Weakness	• **I was so weak, like I couldn't hardly even go to the bathroom without feeling like I was going to pass out**…I’m still kind of weak, I’m still not myself completely, **I’m still a little weak**. • **I felt weak**…The only places I could go was my bed and the bathroom without getting too weak.

The 13 symptoms most reported during concept elicitation (as shown in Table 2) had already been included in the initial version of the ACSD. Only three symptoms mentioned by more than 25% of the sample were not included on the initial version of the ACSD: fever (although “feeling feverish” is included on the ACSD), “brain fog” (which was not a known symptom of COVID-19 at the time the ACSD was first drafted), and dizziness. Participants were also asked which symptoms had been most bothersome (Table 2). All symptoms reported to be “most bothersome” by more than 10% of the sample had already been included on the initial version of the ACSD.

Saturation was examined to assess whether the sample size could be considered sufficient for the study purpose. All symptoms included on the ACSD were reported by participants within the first three interviews. All symptoms reported by 50% or more of the sample were reported within the first three interviews, and all symptoms reported by 10% or more of the sample were reported within the first seven interviews. No new symptoms were reported during the final two interviews, suggesting saturation was reached.

### Cognitive interviews

The first 13 interviews (phase 1) were conducted with the initial version of the ACSD. Based on review of data from phase 1, revisions were made to clarify the recall period, four symptom items, and the global items. These edits were included in the revised version of the ACSD, which was evaluated with 17 interviews in phase 2 (Fig. 1).

All participants (n = 30; 100%) reported that the questionnaire was clear. Participants generally understood the symptom checklist as intended without difficulty, and no differences in comprehension were noted by education level. Table 4 presents example quotations of participant interpretations of each item. Four items on the initial version were revised based on feedback from phase 1, and the revised version was debriefed in phase 2. For example, the item assessing “shortness of breath or difficulty breathing at rest or with activity” in the initial version of the ACSD was simplified to “shortness of breath or difficulty breathing” in the revised version. In addition, the items on taste and smell were shortened from “I have loss of taste/smell” to “loss of taste/smell” for simplicity and consistency with the format of the other items. The item assessing “other COVID-related symptoms” included an open-text field where respondents could write the name of a symptom. This item was removed to simplify the questionnaire for future electronic administration.

**Table 4:** Cognitive Interview Results for the Symptom Checklist of the ACSD

Item on Initial Version (First 13 interviews)	Item on Revised Version (Last 17 interviews)	Selected Quotations Demonstrating Patients’ Interpretations of Each Item
Cough	Cough	Have I had a cough in the past 24 hours.
Shortness of breath or difficulty breathing at rest or with activity	Shortness of breath or difficulty breathing	Initial version of the ACSD: It just means is it hard to breathe when you’re sitting or lying, or you’re walking or moving around, do you have a hard time taking a breath. Initial version: I definitely think my responses would have been different if you had separate “at rest” versus “with activity,” but “shortness of breath” versus “difficulty breathing” I don't think would have changed it at all. Revised version: It’s asking if I have had any issues with my breathing.
Feeling feverish	Feeling feverish	Like I know personally when I have a fever, I always have like burning in my eyes or chills, or like my face is really flushed, so when I read feeling feverish, I think about the symptoms I have when I have a fever.
Chills	Chills	Like when you just feel like you’re freezing for no reason, shaky and cold.
Fatigue (low energy)	Fatigue (low energy)	Not having the energy to do what I would normally do, being at home, like housework, that kind of thing.
Body pain or muscle pain or aches	Body pain or muscle pain or aches	Did you have any aches or pains.
Diarrhea	Diarrhea	If I’m having some diarrhea, soft stool, uncontrolled stool, something like that.
Nausea	Nausea	If you feel like you’ve got to throw up.
Vomiting	Vomiting^1^	Have I actually thrown up anything from my stomach.
Headache	Headache	Have you had any pain in your head in the past 24 hours.
Sore throat	Sore throat	Have I experienced any sore throat or irritation of the throat in the last 24 hours.
Nasal obstruction or congestion (stuffy nose)	Nasal obstruction or congestion (stuffy nose)	If I’m congested, stuffy.
Nasal discharge (runny nose)	Nasal discharge (runny nose)	Have you experienced any runny nose in the last 24 hours.
I have a loss of taste	Loss of taste^1^	I would say that would mean either you can’t taste the food at all or you don't have your normal sense of taste.
I have a loss of smell	Loss of smell^1^	Have I had loss of smell in the past 24 hours.

^1^Four changes to the ACSD are recommended for future research: dropping the item assessing vomiting, adding items assessing brain fog and dizziness, and using 4-point response scales (i.e., absent, mild, moderate, severe) for loss of taste and smell rather than dichotomous responses (i.e., yes, no)

When participants were asked if they experienced any symptoms of COVID-19 that were missing from the questionnaire, only 12 participants (40%) suggested adding symptoms to the questionnaire. Three additions were suggested by more than one respondent, brain fog (n = 5; 17%), burning in the nose (n = 2; 7%), and dizziness/lightheadedness (n = 2; 7%).

The initial version of the ACSD contained two recall periods. The global items asked participants to answer based on their symptoms “today,” while the symptom checklist asked about symptoms “during the past 24 hours.” This inconsistency led to confusion for some participants. To improve clarity in the revised version, the recall period for the global items was changed to “the past 24 hours” to match the symptom checklist. All 17 participants who completed the revised version interpreted the recall period for the symptom checklist and global items as intended.

Participants were asked about the clarity and appropriateness of the symptom checklist response options, which were absent, mild, moderate, and severe. All participants reported that the response options were clear and appropriate for all symptoms. Participants who had experienced a loss of smell or taste were asked about the appropriateness of the dichotomous response options for the two items assessing these symptoms. Of the 20 participants who experienced loss of taste, 13 agreed that dichotomous response options were appropriate for this symptom, while seven reported that there were varying degrees of loss of taste that were not captured by these response options. Of the 22 participants who experienced loss of smell, 15 agreed with the dichotomous response options, while seven reported experiencing varying degrees of loss of smell. It should be noted that all participants were able to answer these two items as intended, even if the response options did not capture varying levels of severity in loss of taste and smell.

The 13 respondents in phase 1 were not asked about the global items, but these items were discussed with the 17 participants in phase 2. The four global items in the revised version of the ACSD assessed (1) overall symptom severity, (2) general physical health, (3) overall symptom change, and (4) return to usual (pre-COVID-19) health in the past 24 h. Items 1, 2, and 4 were easy to complete and understood as intended by all participants. Although all participants reported that global item 3 (“overall change in your COVID-19 symptoms over the past 24 hours”) was easy to answer, 5 of the 17 participants reported thinking about symptom change over time periods longer than 24 h. All five of these participants reported using the 24-h recall period correctly for the other parts of the ACSD, including the symptom checklist.

### ACSD revisions following the interviews (version recommended for future research)

Based on the qualitative results, four edits to the ACSD are recommended for future research. First, an item assessing “brain fog” may be added. Brain fog was reported by 8 of 30 participants during the concept elicitation discussions, including one who considered it the most bothersome symptom. Of the eight participants reporting brain fog, five were still in the acute phase of COVID-19 (i.e., symptom onset < 30 days prior to the interview). Four of the eight participants spontaneously used the term brain fog, while two others used a different term initially but endorsed the term brain fog when it was introduced by the interviewer. The other two participants used a term other than brain fog for this symptom (“foggy” and “tend to forget things”), and this term was not introduced by the interviewer. After completing the ACSD, this was the symptom that respondents most commonly suggested adding to the questionnaire (n = 5). Second, an item assessing dizziness may be added. This symptom was reported by 8 of 30 participants, all of whom used the terms “dizzy” or “dizziness.” After completing the ACSD, two participants suggested adding dizziness to the questionnaire.

A third revision would be to drop the item assessing vomiting. Of the symptoms included in the initial version of the ACSD, this one was most rarely experienced by participants (only 4 of 30). The more common gastrointestinal symptoms of nausea and diarrhea are assessed by other items. The fourth revision would be to change the response scale for the items assessing loss of taste and loss of smell. In the initial version of the ACSD, these items had dichotomous “yes/no” response options. However, in the qualitative interviews, a substantial number of participants reported that severity of these two symptoms varied. Therefore, it would be useful for some patients to rate these two items on the 4-point scale used for the other symptoms included in the diary (i.e., absent, mild, moderate, severe). The version of the ACSD recommended for future research, which includes these four revisions, is presented in Appendix B.

## Discussion

Results of these qualitative interviews support the content validity of the ACSD. The 13 symptoms most commonly reported by participants with COVID-19 during the concept elicitation were included in the initial version of the ACSD. All symptoms reported to be most bothersome by more than 10% of the sample are also included in the instrument. These concept elicitation results suggest that the ACSD assesses symptoms that are relevant and important to individuals with COVID-19.

After participants completed the ACSD, they consistently reported that the questionnaire was clear and easy to complete. All symptom items were interpreted as intended by the great majority of the sample. Respondents also said the instructions and response options were clear, and these were interpreted as intended. The 17 participants completing the revised version of the ACSD had no difficulties with the recall period. In addition, global items assessing overall symptom severity, general physical health, and whether the patient has returned to usual pre-COVID-19 health were easy to complete and understood as intended. Overall, these data suggest the ACSD is clear, comprehensible, easily completed, and interpreted as intended.

This qualitative study should be considered the first step in the validation of the ACSD. While the instrument is clear and relevant to patients, current results suggest that the addition of two more symptoms (brain fog and dizziness) and two other edits can be explored in future research. In addition, research with larger samples will be necessary to examine measurement properties such as reliability, validity, and responsiveness to change. Further analyses are also needed to develop the scoring approach and identify the degree of change in these items that can be considered clinically meaningful.

Despite encouraging results, limitations of some items should be considered. Three symptom items combine multiple terms: “shortness of breath or difficulty breathing,” “body pain or muscle pain or aches,” and “nasal obstruction or congestion (stuffy nose).” While some participants said the components within each item were synonymous, others perceived possible differences. For example, most participants (n = 22; 73%) said nasal obstruction, congestion, and stuffy nose were synonymous, but seven participants (23%) noted differences. Although some respondents perceived differences, it was decided not to separate the components of these combination items since the terms in these three items are usually assessed as a single symptom in clinical settings, and the questionnaire was designed to mirror the typical clinical assessment approach. Still, this issue should be considered as questionnaire development continues. If respondents perceive differences among terms combined for a single item, the item could be a double-barreled question, which could affect validity and responsiveness [29].

One of the four global items may require additional consideration. While global items 1, 2, and 4 were understood as intended by all participants, the recall period of global item 3 (“overall change in COVID-19 symptoms over the past 24 hours”) was misinterpreted by several participants. In the final version of the diary, “past 24 hours” has been bolded in an effort to ensure that respondents notice this recall period in the future. In addition, in a clinical trial setting where patients complete the diary daily, this misunderstanding of the recall period may not have occurred. Future research on this item may clarify the extent to which respondents accurately use this recall period.

Other potential limitations stem from the disease itself. Our understanding of COVID-19 is evolving as more research is conducted and published. For example, when the ACSD was first drafted early in the pandemic, it was not widely known that brain fog was a common symptom. Based on the current study, however, brain fog appears to be a useful addition to the symptom checklist. In addition, the disease itself is changing as new variants emerge, possibly with different symptom profiles. Some ACSD symptom items may be more or less relevant with future variants. As our knowledge of COVID-19 continues to evolve, it is possible that further questionnaire revisions may be necessary.

The possibility of recall bias should also be acknowledged. Participants varied widely with regard to the time elapsed from symptom onset to the interview date. While 13 of the participants were interviewed within two weeks of symptom onset, five were interviewed more than 30 days after experiencing their first symptom. It could have been challenging for some of these participants with longer durations to remember details of their initial days with COVID-19. However, all were required to have had symptoms within seven days of being screened for the study, and there were only two participants who did not currently have symptoms at the time of the interview.

## Conclusions

COVID-19 is associated with a wide range of symptoms, as indicated by the diverse list of symptoms in Table 2. Given the heterogeneous experience of this disease, it would not be feasible to administer a questionnaire that includes all possible symptoms. However, current results indicate that the ACSD captures the most common and bothersome symptoms of COVID-19. The questionnaire appears to be quite comprehensive, while balancing the need for brevity and practicality in clinical trial symptom assessment. Overall, the current qualitative results suggest that the ACSD is a useful PRO measure for assessing symptoms in adults with COVID-19. Future quantitative research is needed to examine the questionnaire’s reliability, validity, and responsiveness to change.

### Supplementary Information

**Additional file 1**. Initial Version of the ACSD.

## Data Availability

The datasets used and/or analyzed during the current study are available from the corresponding author on reasonable request.

## Abbreviations

ACSD: ACTIV-2 COVID-19 Symptom Diary
ACTIV-2: Accelerating COVID-19 Therapeutic Interventions and Vaccines-2
COVID-19: Coronavirus disease 2019
FDA: Food and Drug Administration
PRO: Patient-reported outcome
SARS-CoV-2: Severe acute respiratory syndrome coronavirus 2

## References

[1] Sheikhi K, Shirzadfar H, Sheikhi M (2020). A review on novel coronavirus (COVID-19): symptoms, transmission and diagnosis tests. Trop Med Infect Dis.

[2] Velavan TP, Meyer CG (2020). The COVID-19 epidemic. Trop Med Int Health.

[3] Yap C, Ali A, Prabhakar A, Prabhakar A, Pal A, Lim YY (2021). Comprehensive literature review on COVID-19 vaccines and role of SARS-CoV-2 variants in the pandemic. Ther Adv Vaccines Immunother.

[4] Beigel JH, Tomashek KM, Dodd LE, Mehta AK, Zingman BS, Kalil AC (2020). Remdesivir for the treatment of COVID-19—final report. N Engl J Med.

[5] Gottlieb RL, Nirula A, Chen P, Boscia J, Heller B, Morris J (2021). Effect of bamlanivimab as monotherapy or in combination with etesevimab on viral load in patients with mild to moderate COVID-19: a randomized clinical trial. JAMA.

[6] Korley FK, Durkalski-Mauldin V, Yeatts SD, Schulman K, Davenport RD, Dumont LJ (2021). Early convalescent plasma for high-risk outpatients with COVID-19. N Engl J Med.

[7] Merck Sharp & Dohme Corp. Efficacy and Safety of Molnupiravir (MK-4482) in Non-Hospitalized Adult Participants With COVID-19 (MK-4482-002) (NCT04575597): ClinicalTrials.Gov (Last update posted: November 4, 2021; Recruitment status: Active, not recruting) https://clinicaltrials.gov/ct2/show/NCT04575597. Accessed December 7, 2021.

[8] Nhean S, Varela ME, Nguyen YN, Juarez A, Huynh T, Udeh D (2021). COVID-19: A review of potential treatments (corticosteroids, remdesivir, tocilizumab, bamlanivimab/etesevimab, and casirivimab/imdevimab) and pharmacological considerations. J Pharm Pract.

[9] Aiyegbusi OL, Calvert MJ (2020). Patient-reported outcomes: central to the management of COVID-19. Lancet.

[10] Wong AW, Shah AS, Johnston JC, Carlsten C, Ryerson CJ (2020). Patient-reported outcome measures after COVID-19: a prospective cohort study. Eur Respir J.

[11] Food and Drug Administration (FDA) (May 2020) COVID-19: Developing Drugs and Biological Products for Treatment or Prevention—Guidance for Industry. U.S. Department of Health and Human Services, Center for Drug Evaluation and Research (CDER), Center for Biologics Evaluation and Research (CBER). Silver Spring, MD; 18 p

[12] Food Drug Administration (FDA) (2009). Guidance for industry patient-reported outcome measures: use in medical product development to support labeling claims. Fed Regist.

[13] Patrick DL, Burke LB, Gwaltney CJ, Leidy NK, Martin ML, Molsen E (2011). Content validity–establishing and reporting the evidence in newly developed patient-reported outcomes (PRO) instruments for medical product evaluation: ISPOR PRO good research practices task force report: part 1–eliciting concepts for a new PRO instrument. Value Health.

[14] Patrick DL, Burke LB, Gwaltney CJ, Leidy NK, Martin ML, Molsen E (2011). Content validity–establishing and reporting the evidence in newly developed patient-reported outcomes (PRO) instruments for medical product evaluation: ISPOR PRO good research practices task force report: part 2–assessing respondent understanding. Value Health.

[15] Powers JH, Guerrero ML, Leidy NK, Fairchok MP, Rosenberg A, Hernandez A (2016). Development of the Flu-PRO: a patient-reported outcome (PRO) instrument to evaluate symptoms of influenza. BMC Infect Dis.

[16] Ramakrishnan S, Nicolau DV, Langford B, Mahdi M, Jeffers H, Mwasuku C (2021). Inhaled budesonide in the treatment of early COVID-19 (STOIC): a phase 2, open-label, randomised controlled trial. Lancet Respir Med.

[17] Richard SA, Epsi NJ, Pollett S, Lindholm DA, Malloy AMW, Maves R (2021). Performance of the inFLUenza Patient-Reported Outcome Plus (FLU-PRO Plus) instrument in patients with coronavirus disease 2019. Open Forum Infect Dis.

[18] Chew KW, Moser C, Daar ES, Wohl DA, Li JZ, Coombs R, et al (2021) Bamlanivimab reduces nasopharyngeal SARS-CoV-2 RNA levels but not symptom duration in non-hospitalized adults with COVID-19. medRxiv.

[19] Eli Lilly and Company, AIDS Clinical Trials Group, Brii Biosciences Limited, AstraZeneca, Sagent Pharmaceuticals, Synairgen Research Ltd., et al. ACTIV-2: A Study for Outpatients With COVID-19 (NCT04518410): ClinicalTrials.Gov (Last update posted: December 14, 2021; Recruitment status: Recruiting). Available from: https://clinicaltrials.gov/ct2/show/NCT04518410. Accessed December 7, 2021

[20] LaVange L, Adam SJ, Currier JS, Higgs ES, Reineck LA, Hughes EA (2021). Accelerating COVID-19 therapeutic interventions and vaccines (ACTIV): designing master protocols for evaluation of candidate COVID-19 therapeutics. Ann Intern Med.

[21] Rothman M, Burke L, Erickson P, Leidy NK, Patrick DL, Petrie CD (2009). Use of existing patient-reported outcome (PRO) instruments and their modification: the ISPOR good research practices for evaluating and documenting content validity for the use of existing instruments and their modification PRO task force report. Value Health.

[22] Food and Drug Administration (FDA) (2019) Patient-Focused Drug Development: Methods to Identify What Is Important to Patients—Guidance for Industry, Food and Drug Administration Staff, and Other Stakeholders. U.S. Department of Health and Human Services, Center for Drug Evaluation and Research (CDER), Center for Biologics Evaluation and Research (CBER). Silver Spring, MD; 45 p

[23] Zhou F, Yu T, Du R, Fan G, Liu Y, Liu Z (2020). Clinical course and risk factors for mortality of adult inpatients with COVID-19 in Wuhan, China: a retrospective cohort study. Lancet.

[24] Centers for Disease Control and Prevention (CDC) (December 14, 2021) Demographic trends of COVID-19 cases and deaths in the US reported to CDC. https://covid.cdc.gov/covid-data-tracker/#demographics. Accessed July 8, 2021

[25] Beigel JH, Bao Y, Beeler J, Manosuthi W, Slandzicki A, Dar SM (2017). Oseltamivir, amantadine, and ribavirin combination antiviral therapy versus oseltamivir monotherapy for the treatment of influenza: a multicentre, double-blind, randomised phase 2 trial. Lancet Infect Dis.

[26] Beigel JH, Manosuthi W, Beeler J, Bao Y, Hoppers M, Ruxrungtham K (2020). Effect of oral oseltamivir on virological outcomes in low-risk adults with influenza: a randomized clinical trial. Clin Infect Dis.

[27] Hsieh HF, Shannon SE (2005). Three approaches to qualitative content analysis. Qual Health Res.

[28] Food and Drug Administration (FDA) (2022) Patient-Focused Drug Development: Methods to Identify What Is Important to Patients—Guidance for Industry, Food and Drug Administration Staff, and Other Stakeholders. U.S. Department of Health and Human Services, Center for Drug Evaluation and Research (CDER), Center for Biologics Evaluation and Research (CBER). Silver Spring, MD; 39 pp

[29] Menold N (2020). Double barreled questions: an analysis of the similarity of elements and effects on measurement quality. J Off Stat.

