# Supplementary material for: Qualitative interviews to evaluate content validity of the ACTIV-2 COVID-19 Symptom Diary (ACSD)
Source: J Patient Rep Outcomes. 2023 Jan 31;7:8. doi: 10.1186/s41687-022-00535-x (PMC9888338; doi:10.1186/s41687-022-00535-x)
Supplement: Supplementary file 1 — Additional file 1. Initial Version of the ACSD. [file 41687_2022_535_MOESM1_ESM.docx]

# Supplemental Material: INITIAL Version of THE ACSD (to be available online)

## SYMPTOMS

1. Overall, how bad are your COVID-19 symptoms TODAY (check one)?

No symptoms  Mild  Moderate  Severe

Very severe

1. Overall, how is your general physical health TODAY (check one)

Poor  Fair  Good  Very good  Excellent

1. Have you returned to your usual (pre-COVID) health today (check one)?

Yes  No

Indicate the severity of your symptoms **DURING THE PAST 24 HOURS**

| **Symptoms** | **Absent** | **Mild** | **Moderate** | **Severe** |
| --- | --- | --- | --- | --- |
| Cough |  |  |  |  |
| Shortness of breath or difficulty breathing at rest or with activity |  |  |  |  |
| Feeling feverish |  |  |  |  |
| Chills |  |  |  |  |
| Fatigue (low energy) |  |  |  |  |
| Body Pain or muscle pain or aches |  |  |  |  |
| Diarrhea |  |  |  |  |
| Nausea |  |  |  |  |
| Vomiting |  |  |  |  |
| Headache |  |  |  |  |
| Sore throat |  |  |  |  |
| Nasal obstruction or congestion (stuffy nose) |  |  |  |  |
| Nasal discharge (runny nose) |  |  |  |  |
| Other COVID related symptom, specify: |  |  |  |  |

I have a loss of taste:  Yes  No

I have a loss of smell:  Yes  No
